# Low Light Image Enhancement Algorithm Based on Detail Prediction and Attention Mechanism

**DOI:** 10.3390/e24060815

**Published:** 2022-06-11

**Authors:** Yanming Hui, Jue Wang, Ying Shi, Bo Li

**Affiliations:** School of Information Science and Engineering, Dalian Polytechnic University, Dalian 116039, China; hym0229@126.com (Y.H.); shiying@dlpu.edu.cn (Y.S.); libolb@dlpu.edu.cn (B.L.)

**Keywords:** detail component prediction model, low-light image enhancement, attention mechanism, HSV color space

## Abstract

Most LLIE algorithms focus solely on enhancing the brightness of the image and ignore the extraction of image details, leading to losing much of the information that reflects the semantics of the image, losing the edges, textures, and shape features, resulting in image distortion. In this paper, the DELLIE algorithm is proposed, an algorithmic framework with deep learning as the central premise that focuses on the extraction and fusion of image detail features. Unlike existing methods, basic enhancement preprocessing is performed first, and then the detail enhancement components are obtained by using the proposed detail component prediction model. Then, the V-channel is decomposed into a reflectance map and an illumination map by proposed decomposition network, where the enhancement component is used to enhance the reflectance map. Then, the S and H channels are nonlinearly constrained using an improved adaptive loss function, while the attention mechanism is introduced into the algorithm proposed in this paper. Finally, the three channels are fused to obtain the final enhancement effect. The experimental results show that, compared with the current mainstream LLIE algorithm, the DELLIE algorithm proposed in this paper can extract and recover the image detail information well while improving the luminance, and the PSNR, SSIM, and NIQE are optimized by 1.85%, 4.00%, and 2.43% on average on recognized datasets.

## 1. Introduction

In recent years, low light image enhancement technology seems extremely significant in the field of computer vision (target detection, target recognition), illumination cameras, family security, medical image segmentation, and automatic driving. A dark light image that delivers blurring details, or a video stream with a weak light backdrop will have a grievous impact on the aforementioned domains. What’s more, the current mainstream algorithms are only based on the improvement of image brightness but ignore the extraction of the original detail information of the image, which greatly reduces the information entropy of the image, and the original image information can not be fully expressed. Therefore, low light image enhancement technology has particularly significant research value, moreover, due to its universality, low illumination images are unavoidable in both life and scientific research, and further analysis of its main characteristics reveals the lack of illumination brightness, which leads to the loss of details, obvious noise and the existence of dark areas locally or even as a whole. This will affect the user experience and the further application of scientific research. The main reason for this is insufficient exposure or insufficient surrounding light during acquisition.

At present, for this practical problem, the main low light image enhancement algorithms can be divided into two categories: one is the traditional methods based on HE, Retinex, HSV of color space transform, wavelet transform, dual-domain decomposition, and so on, and they start from the essence of the images, so no matter what the advance of brightness, nevertheless the restoration of details is just barely satisfactory; the other is the artificial neural network method based on deep learning, and has more advantages in brightness hoist and details recovery than traditional methods. However, they are mostly driven by a large number of high-quality data to train the model, which makes the duties of low light enhancement utterly cumbersome. The DELLIE algorithm proposed in this paper combines the operations of traditional algorithms, such as HSV three channels separation, and is based on the idea of deep learning. Then, the detail component prediction model (DCPM) is proposed, which following DELLIE draws into the fiery attention mechanism in the NLP field, which makes the proposed DELLIE algorithm in this paper not only take into account the essence of the image but also give consideration to the deep learning method. At the same time, the detail component of the image can be fleetly estimated, according to the proposed DCPM, and in this way can enormously abate the time complexity of the network model.

In recent years, in traditional methods, adaptive enhancement algorithms for low illumination images based on wavelet transform have been produced [1], dispersed wavelet transform (DWT) has been used to separate the high- and low-frequency subbands of the image, and then enhance them respectively. The image enhancement algorithm based on dual-domain decomposition [2] decomposes the image into a basic layer and detail layer, to realize the decoupling of contrast improvement and noise suppression. The low illumination image enhancement algorithm is based on multi-scale gradient-domain guided filtering, which performs nonlinear global brightness correction on the brightness component, and proposes a brightness enhancement model for enhancement [3]. Both low-light image enhancement via progressive-recursive network [4] and research on image enhancement algorithm based on convolutional neural network in scotopic vision environment [5] have produced certain effects in the enhancement of dark light images. However, because they only focus on enhancing the contrast of images, although the overall brightness has been significantly improved, the corresponding dark area noises may also be amplified, and local areas may be uneven. They have not made remarkable progress in the processing of details retention. Further research shows that HSV color space has more advantages in adjusting picture details than RGB color space. Therefore, Qin et al. proposed the Retinex structured light image enhancement algorithm in HSV color space, and Zhang et al. proposed the retirement low illumination image enhancement algorithm in HSV space [6,7]. While the color is maintained, the information of the image is also enhanced, which further proves the advantages of HSV color space in image processing, but does not focus on how to retain the detail features of dark image edges to the greatest extent.

With the popularity of deep learning in the field of computer vision, neural networks that are data-driven as the main solution began to enter the field of low-light image enhancement. Tao, Li, et al. proposed LLCNN [8]. Ma, Shiping, et al. proposed a low-light sensor image enhancement algorithm based on the HSI color model [9]. Li, Chongyi, et al. proposed LightenNet: A convolutional neural network for weakly illuminated image enhancement [10]. LiCENt, a fast and lightweight algorithm is proposed [11], and the combination of automatic encoder and convolutional neural network (CNN) is used to train the weak light intensifier. Among them, Zero-DCE and Zero-DCE++ first put forward the idea of the zero-reference curve [12,13]. Paired or unpaired datasets can train the model and achieve good results. The kind algorithm adopts the ablation concept for experiment [14] and establishes a simple and effective network to ignite darkness, based on the Retinex theory. However, due to the absence of any reflectivity and illumination information in the GT image, there will be some deviation between the enhanced image and the GT image. Considering the time-consuming disadvantage of traditional methods in processing a single image [15], RetinexNet proposes a convolution neural network based on Retinex theory, which greatly shortens the processing time. It adopts smoothing processing in the enhancement network, to reduce the image sharpness, which makes the reconstructed image slightly blurred. EnlightenGAN proposes an unsupervised generation countermeasure network [16], which can be trained without low/normal light image pairs. Because it does not use GT diagrams for supervised learning, but only uses the benchmark proposed by it for experiments, the results are the same as KinD, and there is also a certain deviation from GT diagrams. LLNet proposes a method based on depth self-coding to extract image features [17], and appropriately enhance image brightness. TBEFN [18] proposes a two-branch exposure fusion network, which is a new generation and fusion strategy and proposes a two-stage denoising strategy to ensure effective noise reduction. DSLR [19] proposed a new low-light-level image enhancement method, based on the useful characteristics of the Laplacian pyramid, in image and feature space. Based on the rich relationship of high-order residuals in multi-scale structure, LIME [20] finds the maximum values in R, G, and B channels to estimate each pixel separately and refines the initial illumination map by adding a structure to the initial illumination map as the final illumination map. C. Chen et al. [21] introduce an original short exposure low light level image dataset. M. Li et al. [22] consider a noise map and propose an optimization function and a logarithmic transformation free alternating direction minimization algorithm based on the Augmented Lagrange multiplier. Y. Shi et al. [23] present a perceptual unsupervised network, and a self-supervised color consistency model is established through a degenerate estimation algorithm to recover lost colors. B. Xu et al. [24] enhance the performance of the algorithm by combining multiscale Retinex color reproduction contrast constrained adaptive histogram equalization. MTRBNet [25] presents a multibranch topology residual block based on information connection and feedback mechanisms. The above deep learning-based image enhancement algorithms have good enhancement performance, but because of the excessive dependence on high-quality and large numbers of datasets, the real-time performance of the algorithms is affected. Singh et al. proposed two exposure-based recursive histogram equalization methods for image enhancement [26]. Jung, Cheolkon, et al. proposed an effective contrast enhancement method based on dual-tree complex wavelet transform [27]. Schmidt et al. proposed shrinkage fields, a random field-based architecture [28]. Gao et al. implemented Retinex algorithms in HSI (Hue, Saturation, Intensity) color space [29]. Gao, Yin, et al. introduced the local weight correction function to each channel pixel value and the Gaussian kernel of the required scale is calculated [30]. Wu, Yahong et al. explored a weighted L 1-norm regularization according to the similarity measure of non-local patches [31]. SA Priyanka et al. proposed a principal component analysis framework to enhance low-light-level images with decomposed luminance–chrominance components [32].

Given the aforementioned analyses, this paper aims at the limitations on the image details, which occurred to the existing low light image enhancement methods. Firstly, by making the improved DexiNed [33] network framework the foundation of the proposed DELLIE, using the proposed detail component prediction model (DCPM), the operation of estimating the enhancement component will be fulfilled quickly and accurately, based on an assigned input image. Secondly, HSV channel separation is the essential measure that takes the decomposing network as an implement, which goes on segregating the reflection map and the illumination map. Furthermore, it is supplemented by the attention mechanism and the improved loss function. Based on deep learning and the innate character of images, an effective algorithm DELLIE is proposed for low-light image detail enhanced recovery and brightness enhancement.

## 2. Related Work

We will introduce HSV color space and attention mechanism.

### 2.1. HSV Color Space

**Figure 1:**
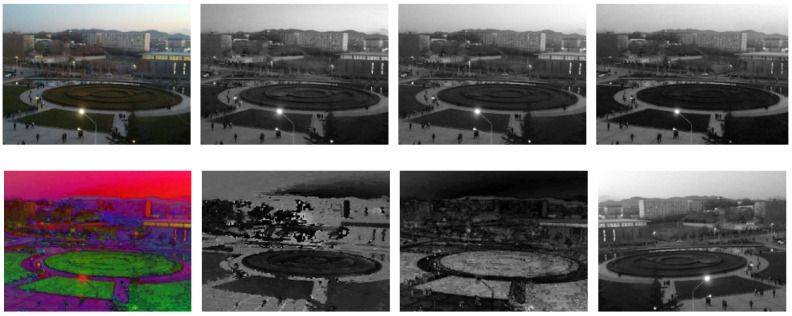
HSV RGB Channel.

With three basic attributes of color hue, saturation and value, HSV color space is a color model for visual perception, which is more in line with human eye characteristics (objective evaluation) than RGB three primary color space, so it is easier to achieve image enhancement results in line with the real situation. Secondly, the three color components of HSV are independent of each other. Adjusting one component alone will not affect the other components, which is more flexible than RGB space, as shown in Figure 1.

(1) $$H=\{\begin{array}{cc} 0^{\circ } & \Delta =0\\ 60^{\circ }\times (\frac{G^{\prime }-B^{\prime }}{\Delta^{\Delta }}\mathrm{mod}6) & ,C_{\max}=R^{\prime }\\ 60^{\circ }\times (\frac{B^{\prime }-R^{\prime }}{\Delta }+2) & ,C_{\max}=G^{\prime }\\ 60^{\circ }\times (\frac{R^{\prime }-G^{\prime }}{\Delta }+4) & ,C_{\max}=B^{\prime } \end{array}$$

(2) $$S=\{\begin{array}{cc} 0 & ,C_{\max}=0\\ \frac{\Delta }{C_{\max}} & ,C_{\max}\neq 0 \end{array}$$

(3) V=Cmax

Through the above formulas, the conversion from RGB image to HSV space can be completed.

### 2.2. Attention Mechanism

Attention mechanism CBAM [34] is extensively applied in natural language processing. Through this mechanism, we can rapidly acquire the target areas of attention, thereupon then receive the focal point of attention, and after that invest more attention to obtain more detailed information about the task concerned. It is discovered in this paper, that even under the constraint of the total loss function, there are trifling structural loss, content loss, and color loss. However, after convolution, a channel attention mechanism (CA) is introduced for high-level features; for low-level features, a spatial attention mechanism (SA) is introduced, and the enhanced feature map obtained in this way will enormously boost the quality of the restored image. Among them, the purpose of using the channel attention mechanism is to make the input image more meaningful by calculating the weights of each channel of the input image through the network, and paying more attention to the channels containing more critical information and less attention to the channels with little important information, thus improving the feature representation capability. Spatial attention aims to enhance the feature representation of key regions, essentially transforming the spatial information in the original image into another space and preserving the key information through a spatial transformation module, generating a weighted mask for each location and weighting the output so as to enhance specific target regions of interest while weakening irrelevant background regions. The overall attention process can be summarized.

(4) $$\begin{array}{l} F^{\prime }=Mc(F)⊗F\\ F^{\prime \prime }=Ms(F^{\prime })⊗F^{\prime } \end{array}$$

where *F*∈*R^C^*^×*H*^^×*W*^ represents intermediate characteristic diagram for input and *F′′* is the final refined output. ⊗ denotes element-wise multiplication. During multiplication, the attention values are broadcasted (copied) accordingly: channel attention values are broadcasted along the spatial dimension, and vice versa. *M_C_*∈*R^C^*^×1^^×1^ represents 1 × 1 Conv channel attention map, *M_s_*∈*R*^1^^×*H*^^×*W*^ represents 2 × 2 Conv spatial attention map and channel attention, as shown Formula (5).

(5) $$\begin{array}{cc} M_{c}(F) & =Sigmoid(MLP(AvgPool(F))+MLP(MaxPool(F)))\\ & =Sigmoid(W_{1}(W_{0}(F_{\mathrm{avg}}^{c}))+W_{1}(W_{0}(F_{\max}^{c}))) \end{array}$$

where *W*_0_∈*R^C/r^^×^^C^*, *W*_1_∈ *R^C^^×^^C/r^* represent the weight value of input and the ReLU activation function. Note that the MLP weights, and they share weights. Spatial attention as shown in Formula (6).

(6) $$\begin{array}{cc} M_{s}(F) & =Sigmoid(f^{7\times 7}([AvgPool(F);MaxPool(F)]))\\ & =Sigmoid(f^{7\times 7}([F_{\mathrm{avg}}^{s};F_{\max}^{s}])) \end{array}$$

*F^s^_*avg*_* ∈ *R*^1^^×*H*^^×*W*^ and *F*^s^_max_ ∈ *R*^1^^×*H*^^×*W*^. Each denotes average-pooled features and max-pooled features across the channel. Those are then concatenated and convolved by a standard convolution layer, producing our 2D spatial attention map. *f*^7×7^ represents a convolution operation with the filter size of 7 × 7.

By innovatively drawing the mature and hot attention mechanism in NLP domain into this algorithm, it is possible to pay preferably attention to the details of the connection between the original image and the restored image.

## 3. Our Approach

The main innovative work of this paper can be summarized as follows. Firstly, this paper proposes a new low-light image enhancement algorithm, called DELLIE, which outperforms other mainstream algorithms in both qualitative and quantitative terms. Second, it proposes the innovative use of basic enhancement pre-processing before the enhancement process. Then this paper proposes a detail component prediction model, which greatly improves the efficiency of recovering image details. Then this paper proposes the decomposition network to improve the efficiency of the separation task of HSV three channels. Subsequently, this paper improves the related loss function and innovatively introduces the hot attention mechanism in NLP to achieve the reconciliation of local and global information in the recovery process.

The principal ideology of the DELLIE algorithm proposed in this paper may be divided into two steps: the first step is distinct from other algorithms. Firstly, the original low light image is going through enhanced preconditioning elementarily, then the heightened detail feature component is obtained through the detail component prediction model (DCPM) proposed in this paper. In the second step, the primitive low light image is separated into three channels: H, S, V, and the enhanced detail feature component, obtained in the first step, is used to enhance the V channel, simultaneously and innovatively introducing the attention mechanism that is common in the field of NLP for local constraints. At the same time, the S channel and H channel are trimmed from the whole situation through the improved loss function. So far, the total DELLIE algorithm has been accomplished.

### 3.1. Algorithm Implementation Process

**Figure 2:**
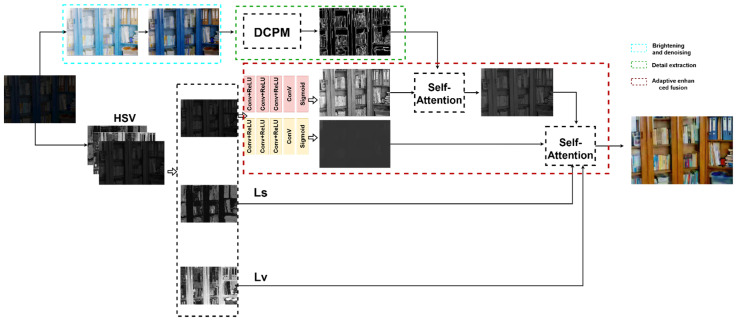
Algorithm flow.

(1) Firstly, the RGB image is converted to HSV color space to gain three independent color channels of the image. According to Retinex theory, the part reflected by the image itself is its inherent attribute. The normal image and the low-light image have identical reflection components, and the V-channel is decomposed into the reflection component and illumination component proposed by DecomNet. (2) Secondly, the primeval low-light image is preprocessed by basic enhancement. Firstly, MSR is used to strengthen the brightness, and Gamma correction is used to suppress the noise. Gamma = 0.65 is selected to suppress noise during the experiment. Afterward, it is transmitted to the proposed detail component prediction model to obtain the enhanced component. (3) Finally, feature fusion is performed under the action of the attention mechanism. Firstly, the enhancement component is used to enhance the V-channel reflection map, and at the same time, the saturation component (S) and hue component (H) are constrained by improved adaptive loss function respectively. Experiments show that the proposed algorithm DELLIE can recover the detailed texture features without excessive brightness or undue contrast heightening, such as in Figure 2.

### 3.2. The Proposed Detail Component Prediction Model

**Figure 3:**
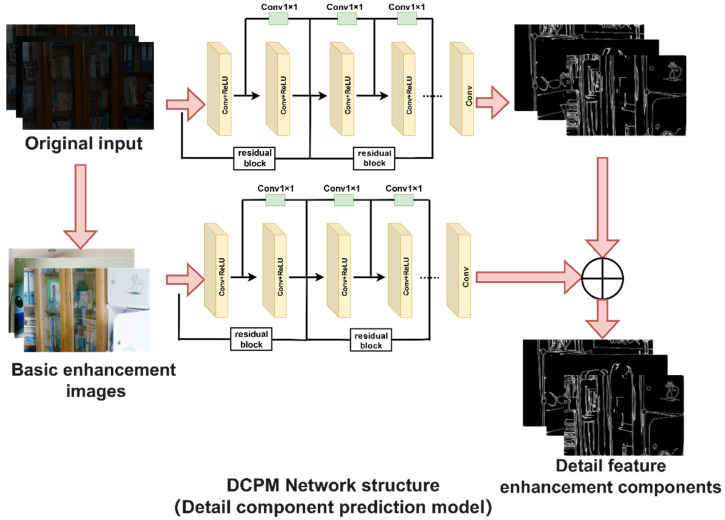
DCPM Network Structure. The DCPM model adopts the network structure Conv+ReLu network layers, and in the last, with 1 × 1 Conv consisting of convolution layer, without pool layer and full connection layer. Among them, Conv+ReLu network has 10 layers; 1 × 1 Conv layer is one layer. The procedure uses only ReLu as the activation function.

(1) Using the current advanced DexiNed network and improving it, the detailed feature maps corresponding to each image are being solved and transformed into HSV channel maps to obtain a V channel map. (2) The low light image, which had gone through basic enhancement, and the V-channel image in (1) are composed of training image pairs, which are transmitted to the DCPM model for training to obtain the detail feature enhancement component. (3) Finally, based on any given low illumination image, the task of accurately generating the corresponding detail feature enhancement component is being achieved. As shown in Figure 3.

In order to better reflect the ability of the proposed detail component prediction model to fully extract the image detail information and to extract and recover more details after the enhanced preprocessing process than without the enhanced preprocessing, the proposed detail component prediction model is presented with a detailed network structure and parameters.

The datasets used are a total of 200 pairs of images that were randomly selected from LOL datasets and Brightening Train datasets, which are for DCPM training. MSR is based on SSR, which has the advantage of maintaining high image fidelity and compressing the dynamic range of the image at the same time. MSR can also achieve color enhancement, color constancy, local dynamic range compression and global dynamic range compression, as shown in (7)–(10).

(7) $$I_{fe}=\sum_{k}^{K}wk\{\log I_{\mathrm{low}}(x,y)-\log[F_{k}(x,y)\cdot I_{\mathrm{low}}(x,y)]\}$$

where *K* is the number of Gaussian center surround functions. When *K* = 1, MSR degenerates to SSR. *F*_k_(*x*, *y*) is centered surround function. *I*_low_ is the input and *I_fe_* is the output. Generally speaking, in order to ensure that the advantages of both high, medium and low scales of SSR are considered, *K* is usually taken to be 3, and there are w1 = w2 = w3 = 1/3. 

At the same time, considering that simple brightening work only into the MSR will largely bring about the amplification of the corresponding noise, the Gamma correction method is first adopted to suppress the noise present, as in the following Equation (8).

(8) $$Ig=C\cdot {I_{fe}}^{\Upsilon }$$

C is called gray scale factor, it is used to stretch the gray scale of the image as a whole, and is usually taken as 1, and in this paper Υ = 0.5. 

In order to improve the extraction ability for image detail information in the algorithm of this paper, the methods of the algorithm in this paper regarding the work of edge texture feature extraction are summarized as follows.

(9) $$I_{\mathrm{dc}p}=l^{n}(W,w^{n})=-\beta \sum_{j\in Y^{+}}\log\sigma (yi=1|I_{g};W,w^{n})-(1-\beta )\sum_{j\in Y^{-}}\log\sigma (yi=0|I_{g};W,w^{n})$$

(10) $$L(W,w)=\sum_{n=1}^{N}\delta^{n}I_{g}l^{n}(W,w^{n})$$

*I_g_*, *I_dcp_* are the results after Gamma and DCPM. *W* is network parameter; *w* is n corresponding parameters. δ is the weight of each scale level. β = |Y −−/|Y + + Y −|, (1 − β) = |Y +|/|Y + + Y −| represent edges and non edges in GT images respectively. In order to strengthen the ability of detail extraction, this paper proposes the idea of iterating the network layer, as shown in (11).

(11) $$LC_{n}(x)=LC_{n-1}(x)+nLC_{n-1}(x)(1-LC_{n-1}(x))$$

$C_{n}\left(X\right)\;\mathrm{and}\;C_{n-1}\left(X\right)$ expresses as each feature layer, n=6 and n≥2. According to the DexiNed algorithm, there are six layers of extracted feature layers, and in this paper, the DELLIE algorithm, after taking full advantage of them, chooses to improve the algorithm by adopting the idea of iteration to iteratively enhance the extraction of feature network layers and fuse them with the previous feature layer. Since the DexiNed algorithm itself does not require previous training or fine-tuning processes, such an iterative process does not cause an increase in processing time; instead, the weight for detailed features is increased in each iteration, so that a more adequate detail confidence is extracted after the completion of the iteration. After the analysis of the experimental results, the detail component prediction model proposed in this paper outperforms the DexiNed algorithm in terms of processing speed and extracting image details. From Figure 3, it is obvious that the extracted image details are more adequate especially after the innovative enhancement preprocessing process is adopted. The specific experimental analysis results can be referred to in Section 4.2: Basic enhancement preprocessing.

#### 3.2.1. DecomNet Network Structure

**Figure 4:**
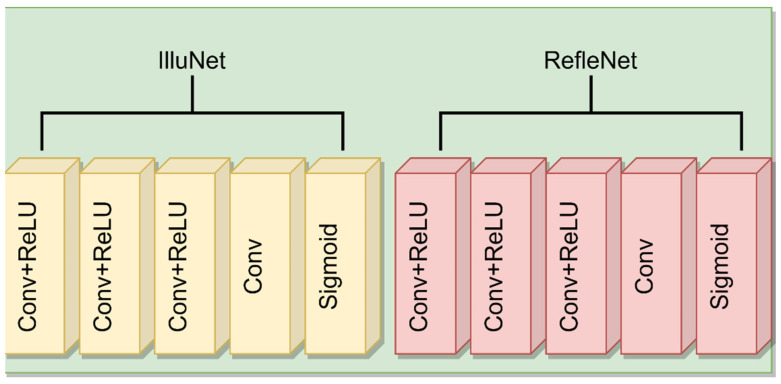
DecomNet network structure.

DecomNet network structure: The network structure of DecomNet is shown Figure 4 below.

DecomNet adopts a five layer network structure, which is composed of three layers of Conv+ReLu, one layer of Conv and sigmoid activation function.

#### 3.2.2. Residual Block Network Structure

**Figure 5:**
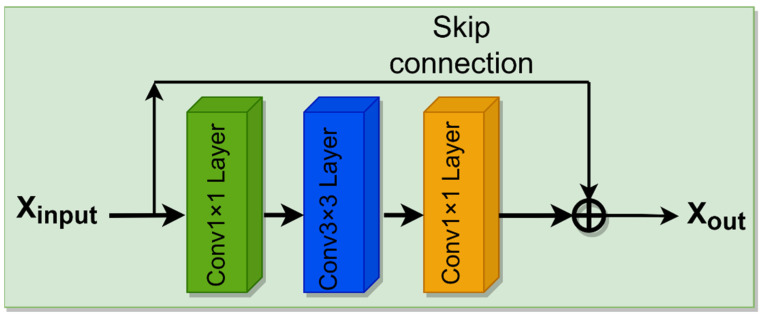
Residual block.

Residual block network structure: The network structure of Residual block network structure is shown in Figure 5 below.

The residual module is modified, and uses firstly 1 × 1 Conv convolution, then this layer is downsampled to reduce the calculation time, and then is 3 × 3 Conv. Finally, it goes through 1 × 1 Conv and skip connection, and channel recovery is carried out for this convolution layer.

### 3.3. The Improved Loss Functions

After HSV decomposition, three channels are obtained. To recover the enhanced image more in line with the subjective and objective standards, this paper improves the loss function: that is, the decomposed three channels are regarded as a whole. While processing the *V* channel, the *S* and *H* channels are pixel-level adaptive to the *V* component, as shown in (12).

(12) $$L_{\mathrm{total}}=w_{h}\sqrt{w_{h}^{p}{\left(\Delta H_{p}\right)}^{2}}+w_{s}\sqrt{w_{s}^{p}{\left(\Delta S_{p}\right)}^{2}}+w_{V}\sqrt{w_{v}^{p}{\left(\Delta V_{p}\right)}^{2}}$$

Considering that the three channels are separated from each other and do not affect each other in the process of low-light image recovery, but it has been proved that if they are not constrained accordingly, the results of the three-channel fusion recovery will result in saturation, chromaticity, and luminance deviating from the human eye perception. Therefore, the three HSV channels are constrained by the corresponding weights, where *w_h_*, *w_s_*, *w_v_*, respectively, represent the weights between the three HSV channels, which are used to constrain the changes between the channels. *w_h_^p^*, *w_s_^p^*, *w_v_^p^* represent the constraints between the pixel points within each channel, so that the constraints between the channels can be carried out after the pixel-level constraints within each channel are completed, so that the local adjustment can be achieved. The global adjustment greatly satisfies the perceptual characteristics of the human eye and is also consistent with the overall scientific rigor. Because of the independence between channels, the relationship between them is nonlinear. In order to minimize the structure loss and content loss of the enhanced image, a pixel-level weighted Euclidean distance method is proposed.

*p* means that the whole is based on the pixel-level, and Δ*H_p_*, Δ*S_p_*, Δ*V_p_* are the losses between the low light image and the real image of each channel respectively; $w_{h}^{p}$, $w_{S}^{p}$, $w_{v}^{p}$ are the weight values of each channel respectively, which are determined by each channel and the sum of the three channels, and considering the overall consistency of the image itself, it is further expressed as (13).

(13) $$L_{\mathrm{total}}={\sum_{c\in \zeta }\left(|{▽}_{x}+{▽}_{y}|\right)}^{2},\zeta =\{H,S,V\}$$

where *c* is the channel and is taken from the three channels *H*, *S*, *V*, and ∇x + ∇y represents the operation of horizontal and vertical gradients respectively. In order to strictly restrict each channel, its weight value is now constrained, as shown in (14)–(16).

(14) $${W_{h}^{p}=(H_{\mathrm{gt}}^{p}+S_{\mathrm{gt}}^{p})(H_{\mathrm{gt}}^{p}+S_{\mathrm{gt}}^{p}+V_{\mathrm{gt}}^{p})}^{-1}+\zeta$$

(15) $${W_{s}^{p}=(S_{\mathrm{gt}}^{p}+V_{\mathrm{gt}}^{p})(H_{\mathrm{gt}}^{p}+S_{\mathrm{gt}}^{p}+V_{\mathrm{gt}}^{p})}^{-1}+\zeta$$

(16) $${W_{V}^{p}=(H_{\mathrm{gt}}^{p}+V_{\mathrm{gt}}^{p})(H_{\mathrm{gt}}^{p}+S_{\mathrm{gt}}^{p}+V_{\mathrm{gt}}^{p})}^{-1}+\zeta$$

ζ is the offset value of limiting weight. The DELLIE algorithm proposed in this paper makes full use of the advantage of the network framework of the improved DexiNed algorithm, which extracts image edge texture information quickly and adopts the idea of iteration to enable it to extract more sufficient edge texture information, and then proposes DCPM. DCPM takes into account the advantages of fast speed and strong extraction ability based on the improved DexiNed algorithm. Then, the classical attention mechanism in the NLP field is innovatively introduced for local adjustment, and the improved loss functions are used to globally restrict the three channels H, S, and V.

## 4. Results

### 4.1. Experimental Conditions and Datasets

**Figure 6:**
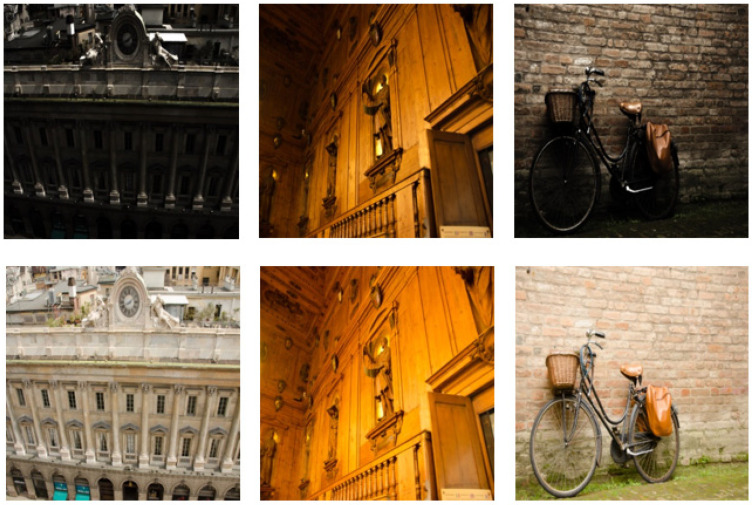
Brightening Train datasets.

**Figure 7:**
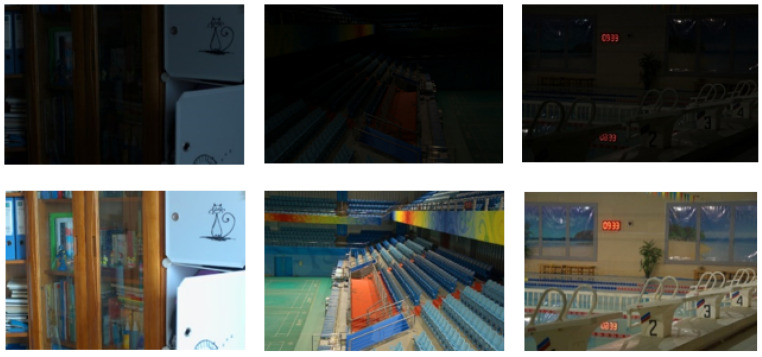
LOL datasets.

All experiments in this paper are carried out in Pycharm. The PyTorch framework is used for deep learning. The memory is 16 G and the graphics card is RTX3080ti. The recognized PSNR, SSIM, NIQE, and AG are used as the objective evaluation indexes of the experimental results, and the LPIPS is used as the subjective evaluation index. To prove the effectiveness of the proposed DELLIE algorithm on different datasets, the datasets of this paper will be selected from recognized LOL datasets, Brightening Train datasets and Mit-Adobe FiveK datasets. The Brightening Train datasets also contain data pairs of dark and normal light images as shown in Figure 6. The LOL datasets include 500 data pairs of very weak light and normal images, obtained by adjusting the exposure time and ISO method as shown in Figure 7. MIT-Adobe FiveK is a database often used by many people doing image enhancement and image retouching research. This database contains 5000 original images in dng format and images retouched by five (A, B, C, D, E) professional retouchers respectively. To demonstrate the randomness and generalization of the experiments, 200 pairs will be randomly selected from these publicly available datasets for this paper. In order to more accurately reflect the efficiency of the model, the ratio of training set, validation set, and test set is set to 6:2:2 in this paper.

To verify the scientificity and realizability of the basic flow of DELLIE algorithm proposed in this paper, the corresponding all-around and multi-angle experimental analyses and evaluations are carried out for each link in the basic flow. For example, (1) analyzes the impact of basic enhancement preprocessing on image detail extraction and (2) analyzes and evaluates the ablation experiment of loss functions in detail recovery, structure adjustment, and content retention; (3) discusses the selection of bias values in the loss functions to determine the range; (4) analyzes the influence of self-attention mechanism on the loss and accuracy of the algorithm; and (5) presents overall result analyses.

### 4.2. Basic Enhancement Preprocessing

**Figure 8:**
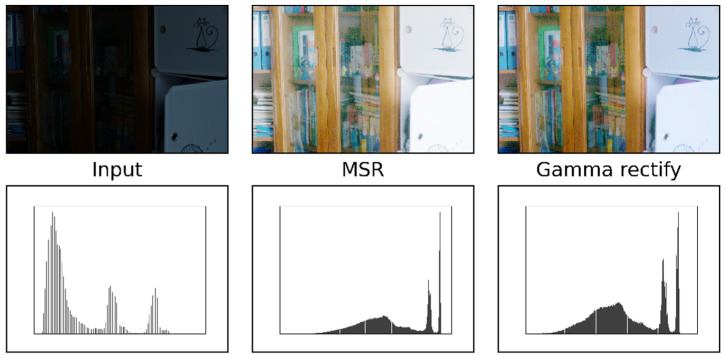
Basic enhancement.

Different from other mainstream algorithms that directly enhance the low light image, to obtain more sufficient detail information map, in the process of obtaining the final enhancement result. As mentioned in the main process of the algorithm proposed in this paper, the proposed algorithm DELLIE differs from other mainstream low-light image enhancement algorithms in that at the beginning of the algorithm, the enhancement pre-processing process is first performed, and the main purpose of this process is to perform the simple task of brightness enhancement and noise suppression without affecting other characteristics of the image itself. The MSR method is used for the task of brightness enhancement, and the Gamma function correction method is used for the task of suppression of noise that may be amplified, and these two tasks are collectively referred to as the enhancement preprocessing process in this paper. From left to right are input, MSR, Gamma correct, and their corresponding gray histogram, as shown in Figure 8.

**Figure 9:**
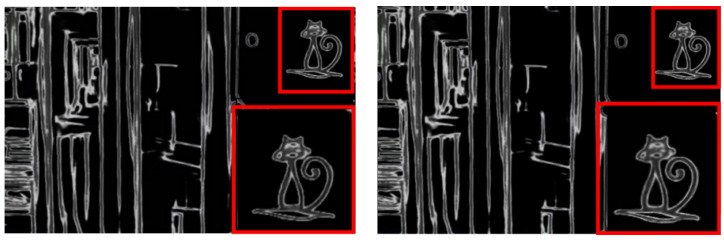
Before Basic enhancement and After Basic enhancement.

In order to prove that more detailed information can be obtained after basic enhancement than without basic enhancement, the experimental results are analyzed, as shown in Figure 9.

**Figure 10:**
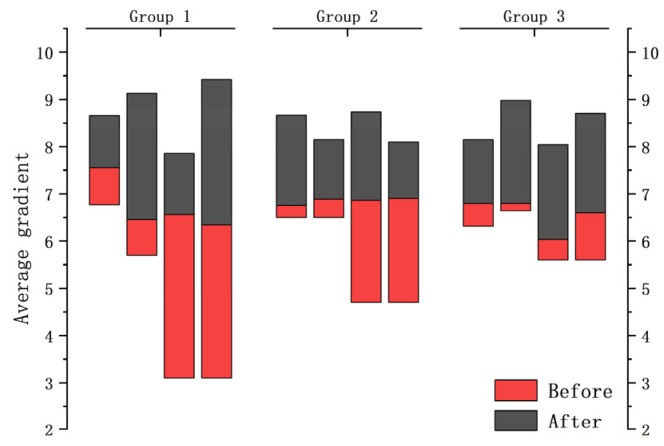
AG of groups.

**Figure 11:**
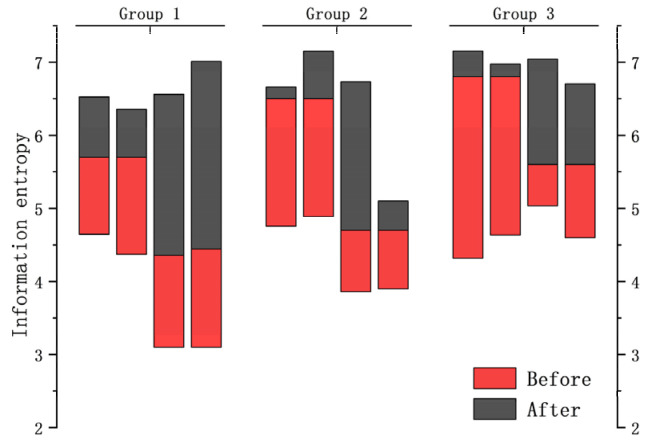
Information entropy of groups.

According to the above figure of After Basic enhancement, the cat pattern in the upper right corner of the bookcase has a smoother outline and more obvious lines. It is proved that more sufficient details are extracted after basic enhancement, which facilitates the subsequent image enhancement and restoration process. In order to prove the generalization ability of DELLIE algorithm, six non-synthetic image pairs are randomly added from the Mit-Adobe FiveK dataset, with the above six images for basic enhancement operation. They are divided into three groups, and four test images in each group are randomly combined images in BrightTrain datasets, LOL datasets, and Mit-Adobe FiveK datasets. The information entropy of an image represents the amount of detailed information that the image contains. The standard value of information entropy of an image is very applicable to the details of the algorithm proposed in this paper; a higher information entropy means that more detailed information is recovered. Now, the comparison of information entropy and AG before and after basic enhancement are shown in Figure 10 and Figure 11 below. 

The experimental results show that the DELLIE algorithm proposed in this paper also has the advantage of a strong ability to extract detailed features and rich information entropy in non-synthetic images. It is further proved that the low-light image can be extracted with more sufficient detailed features after basic enhancement (MSR) and Gamma rectification (GR). Now, the evaluation indexes PSNR, SSIM, and NIQE are compared and analyzed. When MSR = True and GR = True, the left arrow is the comparison of MSR = True and GR = False. The right arrow is a comparison of MSR = False and GR = False, as shown in Table 1. The optimal values for each of these evaluation criteria have been bolded in black in Table 1.

The experimental results show that the information entropy and average gradient of the detail image obtained by basic enhancement are significantly higher, which shows that the image with basic enhancement has richer information and more sufficient details.

### 4.3. Loss Function Ablation Experiment

**Figure 12:**
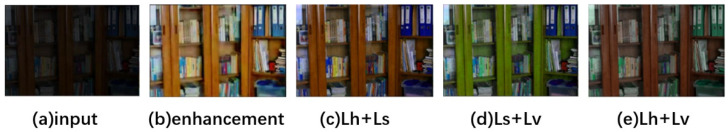
Loss function Ablation Experiment.

In order to verify the advantages of the improved loss functions in detail recovery, structure adjustment, and content retention, an ablation experiment is carried out, as shown in Figure 12.

From the experimental results, we can see that when the V channel is not constrained by the Lv, the image will have too much brightness and noise distortion, which we should try our best to avoid. It can be seen that when the H channel is not constrained by the Lh, the color of the image will be biased, and it can be seen in the bookcase, where the color channel is biased towards a single-color channel. In this experiment, it can be speculated that the color temperature deviation of the restored image is mainly caused by failure to adjust the synchronization constraint with the V channel, that is, the abnormal proportion of blue light in the image. When the S channel is not constrained by the Ls, the image will appear supersaturated or undersaturated. Saturation indicates the purity of the image, which will affect the brightness and color purity of the final result image. Now, the ablation experiment of loss function is compared and analyzed by PSNR, SSIM, and NIQE as shown in Table 2 below. The optimal values for each of these evaluation criteria have been bolded in black in Table 2.

It can be seen from the above figure and table that PSNR, SSIM, and NIQE of the low illumination image are enhanced by the DELLIE algorithm. Although they do not exist in a monotonic trend, they all achieve the best data only at L*_total_* as shown in Table 2. 

### 4.4. Selection of Offset Value

**Figure 13:**
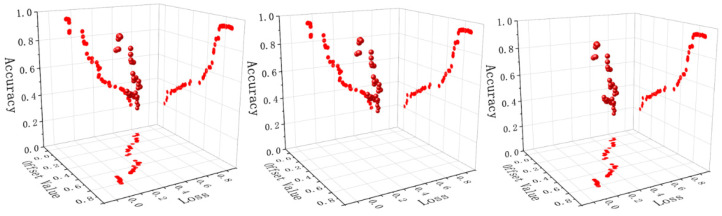
Optimal selection of offset value.

It is found that the different offset values of the loss functions have a certain impact on the loss and accuracy of the algorithm. The first figure shows the impact of different bias values on the whole, and the second and third figures show the impact of different bias values on accuracy and loss respectively, as shown in Figure 13. 

Through the analyses of test results, when the range of offset value is between (0.72568–0.78253), the accuracy of the model is relatively optimal, and the accuracy range is (0.96089–0.96569). The accuracy decreased slightly after offset value being greater than 0.72568, but increased slightly after offset value 0.75416. When the range of offset value is between (0.75416–0.78253), the loss degree of the model is relatively low, and the loss range is (0.16003–0.16067). The offset value between (0.70000–0.80000) is analyzed in detail. The optimal values of loss and accuracy are bold, and the experimental data are shown in Table 3.

### 4.5. Attention Mechanism Ablation Experiment

**Figure 14:**
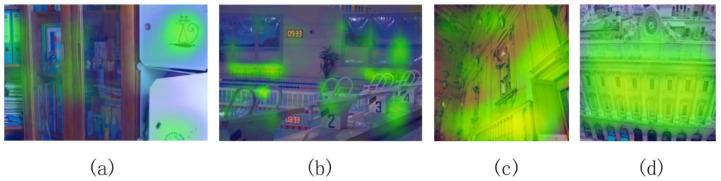

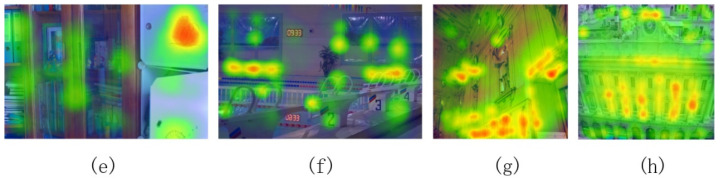
With and without self-attention. Where (**a**–**d**) represent the heat map for image detail capture without adding the attention mechanism to the network, and (**e**–**h**) represent the image heat map with the addition of the attention mechanism.

In order to further prove that the attention mechanism pays attention to the image texture features, and how much attention it pays, now the image heat maps can reflect the degree of attention of the DELLIE algorithm model to a certain feature. The darker the color, the more attention the model pays. Now we analyze the thermodynamic diagram of the experimental data diagrams in this paper. The thermodynamic diagrams without attention mechanism are shown in Figure 14a–d below, and the corresponding thermodynamic diagrams with attention mechanism are shown in Figure 14e–h.

As shown in Figure 14 above, the attention mechanism commonly used in NLP is introduced into the algorithm model, to make the model pay more attention to the feature recovery of details. At the same time, it is combined with the constraints of loss functions, starting from global color recovery and taking into account the retention of local detail features so as to minimize the loss of structure, color, and content.

### 4.6. Overall Result Analysis

**Figure 15:**
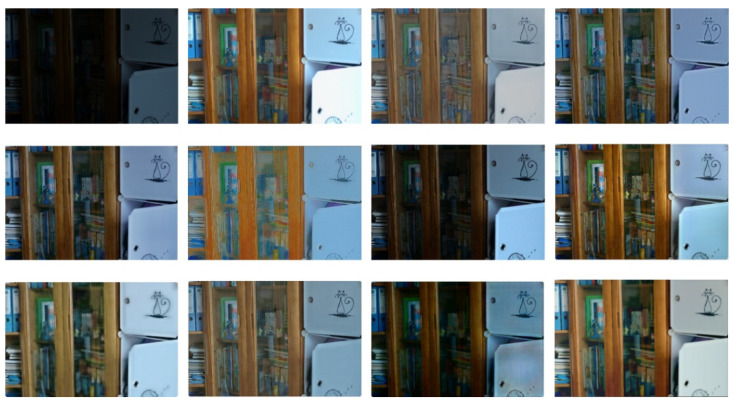
Results of different algorithms on LOL Datasets. From left to right are: Input, GT, KinD [14], Zero-DCE [12], Zero-DCE++ [13], RetinexNet [15], RRDNet [35], EnlightenGAN [16], L LNet [17], TBEFN [18], DSLR [19], Proposed.

**Figure 16:**
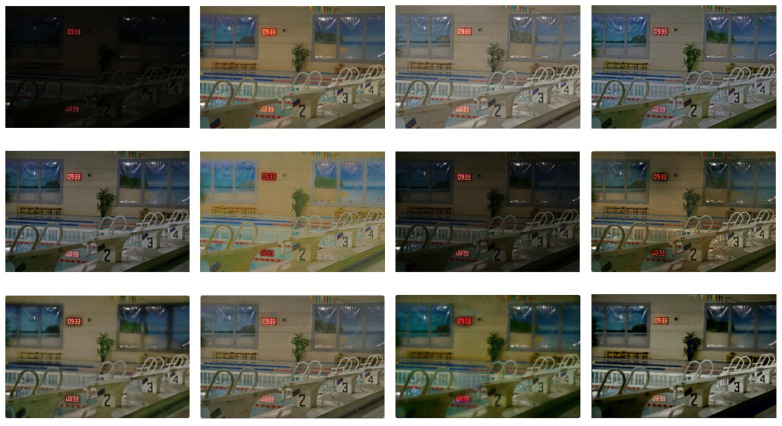
Results of different algorithms on LOL Datasets. From left to right are: Input, GT, KinD [14], Zero-DCE [12], Zero-DCE++ [13], RetinexNet [15], RRDNet [35], EnlightenGAN [16], L LNet [17], TBEFN [18], DSLR [19], Proposed.

**Figure 17:**
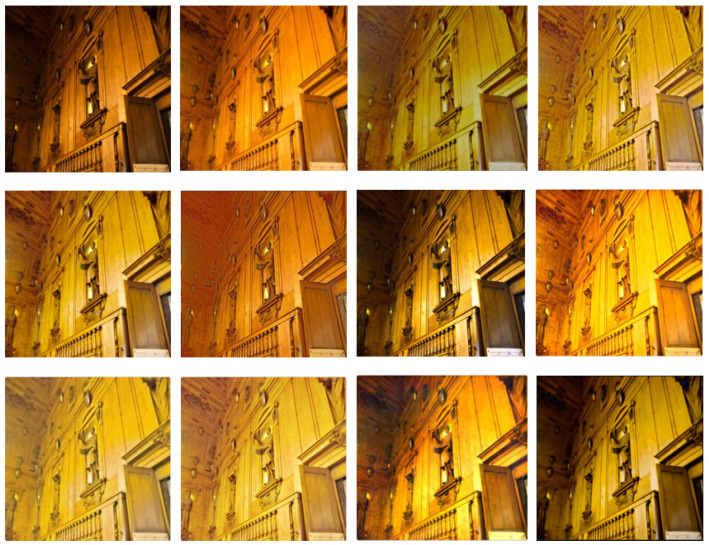
Results of different algorithms on Brightening Train Datasets. From left to right are: Input, GT, KinD [14], Zero-DCE [12], Zero-DCE++ [13], RetinexNet [15], RRDNet [35], EnlightenGAN [16], L LNet [17], TBEFN [18], DSLR [19], Proposed.

**Figure 18:**
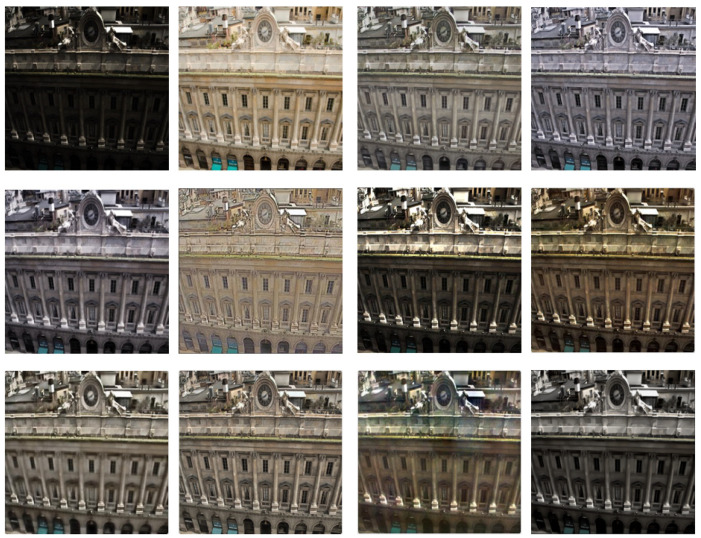
Results of different algorithms on n Brightening Train Datasets. From left to right are: Input, GT, KinD [14], Zero-DCE [12], Zero-DCE++ [13], RetinexNet [15], RRDNet [35], EnlightenGAN [16], L LNet [17], TBEFN [18], DSLR [19], Proposed.

Two recognized datasets and the current mainstream LLIE algorithms are selected to prove the effectiveness of the DELLIE algorithm in this paper. In order to demonstrate the scientific validity and effectiveness of the DELLIE algorithm proposed in this paper, nine mainstream algorithms in the field of low-light image enhancement are selected for quantitative analysis and comparison, and objective indicators recognized in the field, such as PSNR, SSIM, NIQE, Average gradient, Information entropy, LPIPS, etc., are used. Information entropy, LPIPS, etc. from the quantitative point of view conduct a comprehensive quantitative analysis from the subjective and objective evaluation directions. The experimental results of various mainstream algorithms and the DELLIE algorithm proposed in this paper are analyzed more carefully and comprehensively from the qualitative point of view. Both from the qualitative and quantitative perspectives, the superiority of the DELLIE algorithm proposed in this paper can be clearly and objectively demonstrated. From left to right are: Input, GT, KinD [14], Zero-DCE [12], Zero-DCE++ [13], RetinexNet [15], RRDNet [35], EnlightenGAN [16], L LNet [17], TBEFN [18], DSLR [19], Proposed, as shown in Figure 15, Figure 16, Figure 17 and Figure 18 below.

Through the comparative analyses of the experimental results, it can be seen that Zero-DCE, Zero-DCE++, and KinD algorithms, as the mainstream algorithms in the field of low illumination image enhancement, have obvious advantages over other algorithms in terms of brightness improvement and restoration of details. However, the overall tone of the images enhanced by Zero-DCE and Zero-DCE++ are cooler, and the Zero-DCE++algorithm also has some deficiencies in brightness, and the image presents a white effect as a whole. The retinex-net algorithm is too prominent in restoring image texture features, resulting in disharmony of the overall picture, and obvious local noise. The overall brightness of the RRDNet algorithm is dark in the recovery process. In EnlightenGAN, its enhancement operation is based on the generation of a countermeasure network. Due to the lack of paired training data, the brightness enhancement is not clear enough in the enhancement process. The main enhancement effect of LLNet is that the contrast of the image is excessively improved, and the edge of the image is too smooth, which will lose some detailed features. The overall restoration effect of the TBEFN algorithm is very good and close to the GT image. DSLR simply emphasizes the increase of contrast and also ignores the distortion caused by insufficient detail processing. After comparing with the current mainstream algorithms, it is found that the low illumination image enhancement algorithm DELLIE proposed in this paper has obvious advantages in overall and local contrast brightness, and retains a large number of original image details. For example, the outline of the cat in the upper right corner of the first image is more prominent than other algorithms, and the lines are more fully outlined. At the same time, the overall tone is similar to the original image, and there is no obvious imbalance in saturation and contrast. When the second one is restored, the detailed outline of the bench in the upper left corner is also more obvious.

The result analyses of the DELLIE algorithm in Brightening Train datasets are roughly the same as that in LOL datasets. Due to the use of the proposed DCPM model, and the introduction of the attention mechanism, DELLIE extracts more sufficient detail features information and obtains higher information entropy in church and architectural drawings, which makes the restored results clearer at its edges, and the overall effect of the image is more in line with the GT value. To prove the scientificity of the DELLIE algorithm, the recognized PSNR (peak signal to noise ratio), SSIM (structural similarity), and NIQE (natural image quality evaluator) are used as objective evaluation criteria for the experimental results. PSNR is the peak signal ratio, which is an objective standard for evaluating images, and the larger its value, the better; SSIM is a structural similarity, which is an index to measure the similarity between two images, and the larger its value, the better. NIQE uses a multivariate Gaussian model to describe these features. The smaller the value, the more representative the algorithm retains details and reduces artifacts and distortion. For the test images, one of the two datasets is selected, which means that a total of two images form a group, and there are three groups called Test1, Test2, and Test3, as shown in Table 4 below. The optimal values for each of these evaluation criteria have been bolded in black in Table 4.

From the experimental data in Table 4, it can be concluded that the algorithm in this paper has obvious advantages and under-recognized indicators. The optimal value is marked in bold. Considering that the main task of this paper is to restore more sufficient image detail features, the above evaluation standards are general standards. 

**Figure 19:**
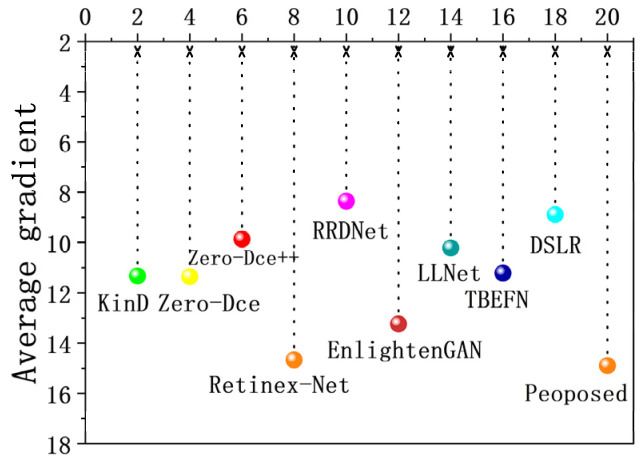
AG of different groups.

**Figure 20:**
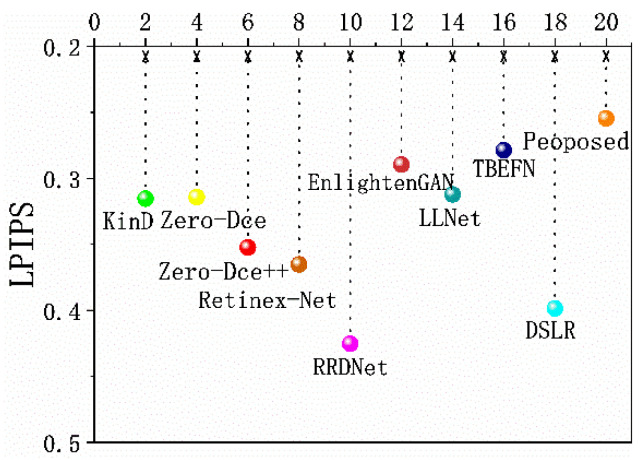
LPIPS of different groups.

To prove the advantages of the DELLIE algorithm over other algorithms in extracting and restoring image details, AG and LPIPS indexes are evaluated for 10 algorithms. AG (Average gradient), that is, the sharpness of the image, reflects the ability to express the comparison of details. The greater its value, the richer the details and the clearer the image. LPIPS (Learned Perceptual Image Patch Similarity), represents the measurement of human visual perception. The smaller the value, the more consistent the sensory effect of the human eyes. The information entropy and average gradient of the image, processed by each algorithm, are shown in Figure 19 and Figure 20. 

From the two charts, it can be analyzed that the algorithm proposed in this paper has more advantages than the mainstream LLIE algorithms in image restoration, and is more in line with the perceptual characteristics of human eyes.

## 5. Conclusions

Through the above experimental analyses, in the process of low-light image enhancement, the details are lost or insufficiently extracted, which could result in insufficient restoration of the original low-light image information, and loss of a large amount of image information entropy. In this paper, the DELLIE algorithm is proposed based on a deep learning artificial neural network. From the analysis of the experimental results, the DELLIE algorithm proposed in this paper has significantly improved the subjective and objective evaluation indexes, when compared with the current mainstream low-light enhancement algorithms, especially in the indicators AG and LPIPS that reflect the ability of the algorithm to extract detailed features, and which have the improvement advantages of 1.61% and 2.45% respectively when compared with the current optimal algorithms. 

The proposed detail component prediction model is mainly used in the experimental process, which can estimate the detail component faster and more accurately based on the given weak light image, and then take it as the enhancement component. Due to the use of the improved DexiNed algorithm, the DCPM proposed in this paper has obvious advantages in processing speed and extracting image details; in the decomposed three channels, the improved loss functions are used for global adjustment, which is a pixel-level constraint. The optimal solution of the offset value is found so that the enhanced image will not have overexposure distortion and saturation excess; it combines the mature spatial and channel attention mechanism in the field of natural language, and pays more attention to local optimization in the recovery process, and greatly reduces the loss of structure, color, and content. Experimental analyses show that the overall accuracy of the algorithm model has been significantly improved after adding this mechanism.

Compared with the current mainstream algorithms, this algorithm achieves the task of low illumination image enhancement, and the extraction of image detail texture features. In the future, due to the practicality of this research, we will conduct further research in the field of face recognition.

## Figures and Tables

**Table 1:** PSNR, SSIM, NIQE of with or not MSR or GR. The optimal value for each of these evaluation criteria has been bolded in black.

	MSR = True			MSR = True			MSR = False		
Epoch	GR = False			GR = True			GR = False		
	PSNR	SSIM	NIQE	↑PSNR↑	↑SSIM↑	↓NIQE↓	PSNR	SSIM	NIQE
600	18.32	0.66	3.75	**18.36**	**0.68**	**3.71**	18.33	0.65	3.73
650	18.58	0.71	3.66	**18.62**	**0.73**	**3.69**	18.59	0.72	3.72
700	18.95	0.77	3.66	**18.96**	**0.79**	**3.63**	18.93	0.75	3.64
750	18.89	0.73	3.70	**18.92**	**0.76**	**3.66**	18.86	0.73	3.68
800	18.93	0.72	3.67	**18.94**	**0.75**	**3.60**	18.87	0.69	3.62

**Table 2:** Loss function Ablation Experiment.

Lh	Ls	Lv	PSNR	SSIM	NIQE
**√**	**√**		19.93	0.71	3.78
	**√**	**√**	19.88	0.75	3.76
**√**		**√**	20.46	0.73	3.82
**√**	**√**	**√**	**20.87**	**0.77**	**3.73**

**Table 3:** Optimal neighborhood variation of 0.7.

Offset Value	Loss	Accuracy
0.71807	0.17311	0.86285
0.72519	0.18095	0.84679
0.72568	0.18116	0.96089
0.75363	0.18615	0.95628
0.75416	0.16047	0.95282
0.75663	0.16061	0.96385
0.76677	0.16003	0.96496
0.78253	0.16067	0.96569

**Table 4:** PSNR and SSIM NIQE of different methods on the Test1, Test2, Test3.

		Test1			Test2			Test3	
Method	PSNR**↑**	SSIM**↑**	NIQE**↓**	PSNR**↑**	SSIM**↑**	NIQE**↓**	PSNR**↑**	SSIM**↑**	NIQE**↓**
KinD [14]	20.86	0.70	3.76	22.31	0.63	3.77	18.66	0.56	3.77
Zero-Dce [12]	14.88	0.52	3.73	14.56	0.48	3.78	14.39	0.52	3.74
Zero-Dce++ [13]	16.75	0.65	3.70	15.32	0.52	3.79	16.98	0.64	3.78
Retinex-net [15]	16.73	0.55	3.68	17.25	0.47	3.75	16.32	0.48	3.74
RRDNet [35]	18.83	0.74	3.77	16.83	0.54	3.73	15.96	0.65	3.76
EnlighenGAN [16]	21.65	0.75	3.74	20.86	0.63	3.76	17.89	0.71	3.75
LLNet [17]	18.34	0.68	3.72	21.32	0.58	3.74	18.62	0.64	3.73
TBEFN [18]	21.56	0.75	3.78	22.33	0.57	3.76	19.35	0.59	3.74
DSLR [19]	20.89	0.74	3.75	21.83	0.61	3.74	18.63	0.57	3.73
Proposed	**21.86**	**0.76**	**3.66**	**22.58**	**0.68**	**3.70**	**20.03**	**0.73**	**3.71**
